# [Corrigendum] Potassium increases the antitumor effects of ascorbic acid in breast cancer cell lines *in vitro*

**DOI:** 10.3892/ol.2026.15494

**Published:** 2026-02-17

**Authors:** Giovanni Vanni Frajese, Monica Benvenuto, Massimo Fantini, Elena Ambrosin, Pamela Sacchetti, Laura Masuelli, Maria Gabriella Giganti, Andrea Modesti, Roberto Bei

Oncol Lett 11: 4224–4234, 2016; DOI: 10.3892/ol.2016.4506

Following the publication of the above article, an interested reader drew to the authors’ attention that the ERK blots shown for the MCF-7 and MDA-MB-231 cell lines in Fig. 3 on p. 4231 were unexpectedly similar, given that the blots were intended to show the results from experiments performed on two different cell lines. Upon asking the authors for an explanation, they were able to provide the raw data underlying the figure, and they realized that the blots correctly shown for the MDA-MD-231 cell line had inadvertently been copied across for the MCF-7 cell line. The corrected version of Fig. 3, now showing the correct ERK blots for the MCF-7 cell line, is shown below. The authors regret that this error occurred during the assembly of Fig. 3, although this did not grossly affect either the results or the conclusions reported in this article. All the authors agree with the publication of this Corrigendum, and thank the Editor of *Oncology Letters* for granting them the opportunity to publish this; furthermore. they apologize to the readership for any inconvenience caused.

## Figures and Tables

**Figure 3.**
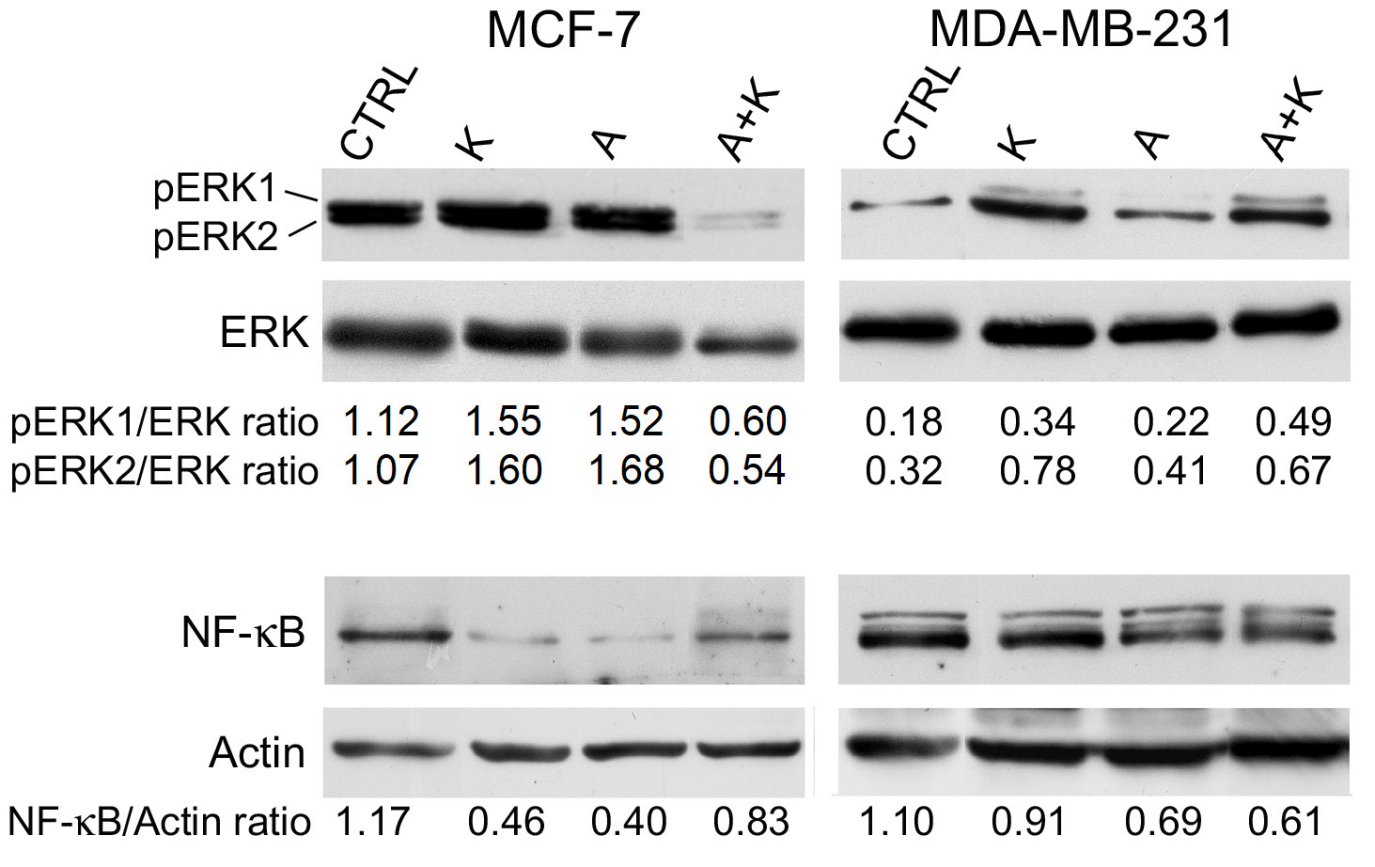
Effect of treatment with A and K, alone or in combination, on pro-survival signaling proteins. Western blotting was performed on MCF-7 and MDA-MB-231 cells treated with A or K, alone or in combination, for 24 h. The levels of phosphorylated ERK1/ERK2 were compared with the total protein levels of ERK, and the ratio values are reported. Quantitative densitometric analysis of the expression levels of nuclear factor-κB, compared with the levels of actin, is provided. The faint higher molecular weight product observed with the NF-κB antibody in the MDA-MB-231 cells may be due to non-specific reactions in this cell line. CTRL, culture medium; K, potassium; A, ascorbic acid; ERK, extracellular signal-regulated kinase; p, phosphorylated; NF-κB, nuclear factor-κB.

